# The effects of subliminal or supraliminal sadness induction on the sense of body ownership and the role of dissociative symptoms

**DOI:** 10.1038/s41598-021-01039-2

**Published:** 2021-11-15

**Authors:** Franziska A. Schroter, Bianca A. Günther, Petra Jansen

**Affiliations:** Faculty of Human Sciences, University of Regensburg, Regensburg, Germany

**Keywords:** Psychology, Psychiatric disorders

## Abstract

Previous research has shown that emotions can alter our sense of ownership. Whether this relationship is modulated by differences in emotion experience and awareness, however, remains unclear. We investigated this by comparing the susceptibility to the rubber hand illusion (RHI) between participants who were either exposed to a low-arousing emotion induction (sadness) or placed in a neutral control group. Several factors that might influence this relationship were considered: dissociative symptoms were included to observe if a sadness induction led to a higher RHI score in participants scoring high in dissociation, as a result of detached emotion experience. Whether the level of awareness of the emotion mattered was also tested, as subliminal processing was shown to require less focal attention. Therefore, our sample (*N* = 122) was divided into three experimental groups: Sad pictures were presented to two of the three groups differing in presentation mode (subliminal: *n* = 40, supraliminal: *n* = 41), neutral pictures were presented supraliminally to the control group (*n* = 41). Additionally, the effects of slow (3 cm/s) and fast (30 cm/s) stroking, applied either synchronously or asynchronously, were examined as the comforting effects of stroking might interfere with the emotion induction. Results showed that the supraliminal sadness induction was associated with a stronger subjective illusion, but not with a higher proprioceptive drift compared to the subliminal induction. In addition, a stronger subjective illusion after fast and synchronous stroking was found compared to slow and asynchronous stroking. A significant proprioceptive drift was detected independent of group and stroking style. Both slow and synchronous stroking were perceived as more comforting than their respective counterparts. Participants with higher dissociative symptoms were more susceptible to the subjective illusion, especially in the supraliminal group in the synchronous condition. We concluded that individual differences in emotion experience are likely to play a role in body ownership. However, we cannot clarify at this stage whether differences in proprioception and the subjective illusion depend on the type of emotion experienced (e.g. different levels of arousal) and on concomitant changes in multisensory integration processes.

## Introduction

The feeling that the body, bodily sensations and body parts belong to oneself has previously been described as the *sense of body ownership*^1^. It can be investigated using the *rubber hand illusion* (RHI)^2,3^. In this paradigm, the real hand is hidden from view and a rubber hand is placed in front of the participants. Both hands are stroked, for example with a brush, which can elicit the illusion that the rubber hand is actually the participant’s own hand^2^. This illusion can be measured for instance by using a questionnaire or by assessing the *proprioceptive drift*^2^. Proprioceptive drift refers to the phenomenon where a person's estimation of the position of their own hand in space drifts towards the position of the rubber hand. Greater proprioceptive drift is used as an indicator of a successful illusion induction^2,3^.

Studies have provided preliminary evidence that affective factors and emotions may be associated with changes in body ownership. For example, slow affective stroking (3 cm/s) has been found to be perceived by participants as more pleasant and has elicited a stronger illusion than fast stroking (18 cm/s or 30 cm/s)^4–6^. In addition, direct involvement of the emotional system in body ownership has been suggested: An investigation into the relationship between threat and the RHI revealed that threatening the rubber hand with a needle evoked the same pattern of brain activity as threatening the participants’ own hand^7^. It also showed that a higher threat response in the anterior cingulate cortex and the left insular cortex was associated with a stronger illusion^7^. Furthermore, the effects of affective vocalizations, like anger or happiness, on the RHI have been studied in the past: In their first experiment, Engelen et al. compared four groups (angry, happy, non-vocal and no sounds) and found a greater proprioceptive drift following emotional vs. unemotional sounds^8^. A similar effect was found in their second experiment, in which the authors only compared angry, neutral and no sound conditions. The study by Engelen et al.^8^ demonstrated that the RHI intensity seems to be independent of affective valence (negative or positive). Interestingly, all studies that investigated the relationship of emotions and the sense of body ownership focused on high arousing emotions only^9^. More attention should be drawn to the effects of low-arousing emotions.

Regarding low-arousing emotions, as yet unanswered questions are: Does a low-arousing emotion such as sadness show the same effects on body ownership? And if so, are there factors that influence this relationship? A body of research has demonstrated that the way emotions are processed and experienced also has an impact on the sense of body ownership. To name one example, higher scores in an alexithymia questionnaire were associated with a stronger illusion in a study with adolescents^10^. Hence, differences in emotion experience could influence the relationship of emotions and sense of body ownership. For example, a detached emotion experience, as it occurs in dissociation, could be relevant in this context. According to Frijda, emotion experience can vary depending on the situation and the individual^11^. It has been stated that emotion experience involves 1st order and 2nd order experiences^12^. The former refers to a phenomenological experience of the emotion; the latter refers to the awareness of this phenomenological state, allowing us to express, report and memorize an emotional state. The presence of this 2nd order experience is optional. 1st order experience can be directed either towards the self, reflecting an evaluated self or a representation of the bodily self, or towards the world, involving spatial experiences, as in action direction^12^. Lambie and Marcel propose a model of focal attention, which suggests that the 2nd order experience is shaped by three components: the mode of attention which can be analytic or synthetic and immersed or detached; the attentional focus, which can be either on the emotional evaluation or on the planned action; and the direction of attention, which can be focused on the self or on the world^11,12^. In this way, the content of the 1st order phenomenology can become subject to 2nd order awareness. When having an attentional world focus, subjects undergo a haptic perceptual experience when being touched. In contrast, if the focus is on the self, the experience of touch focuses on the tactile bodily sensation, which also includes proprioceptive awareness and the feeling of ownership. Detachment from the emotion in the form of dissociative avoidance could in turn prevent the self-focused experience of the emotion^12^. Detached attentional mode along with a self-focus can lead to depersonalization experiences^11^, which often include symptoms like disembodiment (detachment of the body) and emotional numbing^13,14^. Similarly, detachment with world-focus more likely leads to derealization symptoms^11^. Dissociative symptoms, including depersonalization and derealization have been associated with a stronger RHI in patients with borderline personality disorder^15^ and post-traumatic stress disorder^16^. There is also evidence from another study showing high variance in the illusion strength in patients with dissociative PTSD^17^. This finding indicates that a variable explaining these differences in the relationship of dissociation and the RHI might still be missing. Since it has been argued that dissociative symptoms arise in response to aversive emotions^15,18,19^, the current mood at the time of illusion induction might interact with the strength of the illusion. To test this, we aimed to examine the effect of a negative emotion induction on the RHI strength while accounting for dissociative symptoms.

Another influencing factor on the relationship between emotion induction and the RHI could be the level of emotion experience. Lambie and Marcel suggest that when emotional stimuli are presented very briefly, participants do not have time to engage analytically with the stimuli and consequently their 2nd order awareness of the emotion is reduced^12^. Indeed, subliminally presented emotional stimuli do not alter conscious affect but have been found to lead to similar behavioral changes like supraliminal emotion induction^20^. According to Lambie and Marcel, focal attention is not required for 1st order attention, but it is known to affect it^12^. Accordingly, 2nd order focal attention mechanisms might be less involved in processing subliminal emotional stimuli. In line with this, supraliminal processing has been shown to involve brain regions associated with top–down emotion regulation, attention and consciousness, whereas subliminal processing involves regions that reflect more autonomic emotional states^21^. However, since the distinction between self- and world focus is also present in 1st order experience, ownership processes might still play a role here. Given the differences in affect, cortical activation, and attention between subliminal and supraliminal processing, we asked whether embodiment processes are already present to some extent in the pre-conscious stage of an emotional state.

Finally, a secondary aim of this study was to investigate if the method of illusion induction could also influence the link between emotion and body ownership. As mentioned earlier, different stroking speeds have been found to affect the illusion in different ways. Applying touch that is perceived as gentle excites a special cell type, called C-tactile (CT) nerve fibers. These fibers are sensitive to slow, pleasant touch. They are located mostly in hairy skin, e.g. at the forearm^22,23^, but can also be found in the glabrous skin of the human hand, highlighting the importance of considering stroking speed in rubber hand paradigms^24^. Pleasant touch can also have a comforting effect: A study with 84 females examined the role of touch on feelings of social exclusion and showed that social pain can be reduced by slow, pleasant touch compared to fast, unpleasant touch^25^. Besides, Riemer et al. stress the importance of considering different pleasant or unpleasant stroking styles in studies investigating affective components, as interactions with the induced emotion are possible^26^.

In summary, the main aim of this study was to examine the effects of a low-arousing emotion (in this case sadness) vs. a neutral control condition on the intensity of the rubber hand illusion, while considering factors related to emotion experience, such as dissociative symptoms and the level of awareness of the emotion induction. For this purpose, three experimental groups were implemented: one group saw supraliminally presented sad pictures, another group saw subliminally shown sad pictures, masked by neutral pictures and a third group saw only neutral supraliminally presented pictures. Additionally, slow and fast stroking conditions were added, because slow stroking in the emotion induction condition may have comforting effects^25^ that could reduce the effect of the sadness induction. Moreover, slow and fast stroking were combined with either synchronous or asynchronous stroking, as it is suggested to include control conditions such as asynchronous touch to control expectancy effects^26^.

The following hypotheses were examined:

1. A stronger subjective illusion and proprioceptive drift is expected following the sadness induction compared to a neutral condition^8^.
2. Participants scoring high on a dissociative symptom questionnaire may experience a stronger illusion compared to participants with a lower symptom score^15^. It will also be investigated whether the emotion induction interacts with dissociative symptoms^12,15,18,19^.
3. Supraliminal emotion induction is expected to have the strongest effect on the illusion, followed by subliminal induction, while the lowest illusion intensity is expected in the neutral condition^12,20^.
4. Slow and synchronous touch are assumed to lead to stronger illusion effects than their counter-conditions, fast and asynchronous stroking. At the same time, interactions of stroking speed with the emotion induction condition are expected, as slow stroking might interfere with the sadness induction^25,26^.

## Results

### Sadness induction effects on valence, arousal and dominance

**Figure 1:**
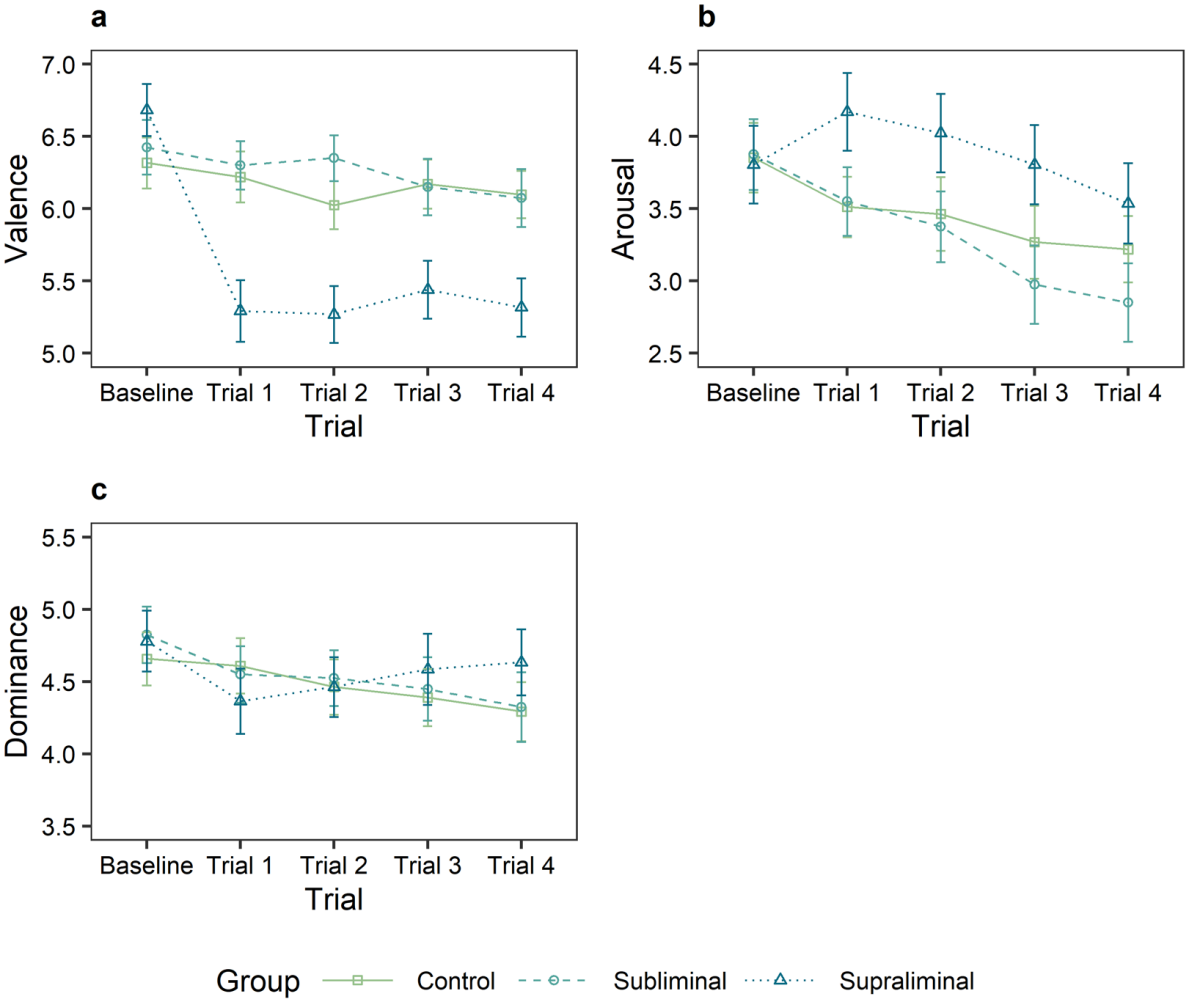
SAM ratings depending on trial and group affiliation. (**a)** Valence ratings, (**b)** arousal ratings, (**c)** dominance ratings. Error bars correspond to the standard error of the mean.

Valence, arousal and dominance ratings were assessed with the self-assessment manikin (SAM)^27^ and analyzed using cumulative link mixed models. The predictors group and trial were entered into the model. For all three variables, non-significant random slopes and fixed effects were dropped, resulting in random intercept models. There was a significant reduction of valence in trial 1 to 4, a reduction of arousal in trial 2 to 4 and a reduction of dominance in trial 1 to 4 compared to the baseline measurement (Table 1, Fig. 1). A main effect of group was found for the valence dimension, in terms of a lower valence in the supraliminal compared to the subliminal group. For valence, there was also a significant interaction between trial and control vs. intervention group. By looking at Tukey corrected post-hoc comparisons, it becomes clear that this interaction is due to significant valence reductions in the supraliminal group compared to the control group (B/T1: *β* = 1.59, *z* = 3.92, *p* = .008; B/T2: *β* = 1.63, *z* = 4.01, *p* = .006; B/T3: *β* = 1.36, z = 3.37, *p* =.054; B/T4: *β* = 1.55, z = 3.83, *p* = .011), while no differences were found between control and subliminal group (B/T1: *β* = 0.01, *z* = 0.04, *p* = 1.00; B/T2: *β* = -0.07, *z* = -0.16, *p* = 1.00; B/T3: *β* = 0.25, z = 0.62, *p* = 1.00; B/T4: *β* = 0.37, z = 0.91, *p* = .999). Moreover, an interaction between trial and subliminal vs. supraliminal group was found, showing a drop in valence in the supraliminal group compared to the subliminal group (Table 1, Fig. 1a). Besides, an interaction between trial and subliminal vs. supraliminal group was found for the variable arousal, showing a stronger decrease in arousal in the subliminal group compared to the supraliminal group (Fig. 1b).

**Table 1:** Final cumulative link mixed models for the dependent variables valence, arousal and dominance.

	Valence					Arousal					Dominance				
	Estimate	*SE*	*z*	*p*	*95% CI*	Estimate	*SE*	*z*	*p*	*95% CI*	Estimate	*SE*	*z*	*p*	*95% CI*
**Fixed effects**															
*Trial*															
B/T1	− 0.84	0.14	− 5.80	** < .001**	− 1.12; − 0.55	− 0.14	0.14	− 1.03	.302	− 0.42; 0.13	− 0.44	0.15	− 3.04	**.002**	− 0.72; − 0.16
B/T2	− 0.92	0.14	− 6.35	** < .001**	− 1.20; − 0.64	− 0.32	0.14	− 2.25	**.024**	− 0.59; − 0.04	− 0.48	0.15	− 3.32	** < .001**	− 0.77; − 0.20
B/T3	− 0.86	0.14	− 5.97	** < .001**	− 1.14; − 0.58	− 0.72	0.14	− 5.00	** < .001**	− 1.00; − 0.44	− 0.50	0.15	− 3.42	** < .001**	− 0.78; − 0.21
B/T4	− 1.00	0.15	− 6.87	** < .001**	− 1.28; − 0.71	− 0.93	0.15	− 6.39	** < .001**	− 1.21; − 0.64	− 0.60	0.15	− 4.13	** < .001**	− 0.89; − 0.32
*Group*															
Con./Int.	− 0.12	0.10	− 1.23	.219	− 0.31; 0.07	0.06	0.13	0.46	.643	− 0.20; 0.32					
Sbl./Spr.	− 0.52	0.17	− 3.03	**.002**	− 0.85; − 0.18	0.38	0.23	1.63	.104	− 0.08; 0.84					
*Trial × group*															
B/T1 × Con./Int.	− 0.34	0.10	− 3.40	** < .001**	− 0.54; − 0.14	0.17	0.10	1.68	.093	− 0.03; 0.36					
B/T2 × Con./Int.	− 0.23	0.10	− 2.34	**.019**	− 0.43; − 0.04	0.12	0.10	1.18	.239	− 0.08; 0.31					
B/T3 × Con./Int.	− 0.32	0.10	− 3.19	**.001**	− 0.51; − 0.12	0.06	0.10	0.56	.574	− 0.14; 0.25					
B/T4 × Con./Int.	− 0.33	0.10	− 3.33	** < .001**	− 0.53; − 0.14	− 0.02	0.10	− 0.16	.870	− 0.21; 0.18					
B/T1 × Sbl./Spr.	− 0.98	0.18	− 5.55	** < .001**	− 1.33; − 0.63	0.47	0.17	2.75	**.006**	0.14; 0.81					
B/T2 × Sbl./Spr.	− 1.04	0.18	− 5.85	** < .001**	− 1.38; − 0.69	0.49	0.17	2.86	**.004**	0.16; 0.83					
B/T3 × Sbl./Spr.	− 0.75	0.17	− 4.27	** < .001**	− 1.09; − 0.40	0.65	0.18	3.74	** < .001**	0.31; 1.00					
B/T4 × Sbl./Spr.	− 0.78	0.17	− 4.48	** < .001**	− 1.13; − 0.44	0.55	0.18	3.14	**.002**	0.21; 0.89					
**Threshold estimates**															
Threshold	− 5.43	0.25	− 21.89		− 5.92; − 4.94	− 2.64	0.22	− 12.20		− 3.07; − 2.22	− 5.30	0.28	− 18.79		− 5.85; − 4.75
Spacing	1.55	0.06	26.34		1.43; 1.66	1.33	0.05	26.19		1.23; 1.43	1.75	0.07	25.72		1.62; 1.89
**Random effects**															
τ_00 participant_	2.09					4.12				4.26					

*B* Baseline, *T1* Trial 1, *T2* Trial 2, *T3* Trial 3, *T4* Trial 4, *Con.* Control group, *Int.*  Intervention, *Sbl*. Subliminal group, *Spr.* Supraliminal group.

### Comforting effects

Cumulative link mixed models with the predictors synchrony, speed and group were computed to assess whether the perceived comfort of touch depended on the stimulation condition and the group. After reducing the variance components, the intercept only model was found to have the best fit. Both within subject main effects, speed and synchrony, turned out to be significant (Table 2). Slow stroking (*Mdn* = 3.00) had a stronger comforting effect than fast stroking (*Mdn* = 2.50) and synchronous stroking (*Mdn* = 3.00) had a stronger effect than asynchronous stroking (*Mdn* = 2.50). A significant interaction between speed and synchrony was found, as well (Tables 2 and 3). The main effect of group was not significant and all further interaction effects were dropped in the model complexity reduction.

**Table 2:** Final cumulative link mixed model for the dependent variable comfort rating.

	Comfort rating				
	Estimate	*SE*	*z*	*p*	*95% CI*
**Fixed effects**					
Speed	− 0.67	0.15	− 4.45	** < .001**	− 0.97; − 0.38
Synchrony	− 0.69	0.15	− 4.59	** < .001**	− 0.99; − 0.40
*Group*					
Con./Int.	0.23	0.12	1.83	.067	− 0.02; 0.47
Sbl./Spr.	0.38	0.22	1.75	.081	− 0.05; 0.80
Speed × synchrony	0.46	0.21	2.17	**.030**	0.04; 0.87
**Threshold estimates**					
Threshold	− 2.41	0.23	− 10.39		− 2.86; − 1.95
Spacing	1.55	0.08	20.59		1.40; 1.70
**Random effects**					
τ_00 participant_	3.36				

*Con*.  Control group, *Int.*  Intervention, *Sbl.* Subliminal group, *Spr.* Supraliminal group.

**Table 3:** Means (standard deviations) / frequencies (relative %) / medians of demographic data and outcome variables.

	Neutral control group	Subliminal sadness group	Supraliminal sadness group
**Demographic data**			
Female	*n* = 27 (65.85%)	*n* = 29 (72.50%)	*n* = 30 (73.17%)
Male	*n *= 14 (34.15%)	*n* = 11 (27.50%)	*n* = 11 (26.83%)
Age	*M* = 21.49 (2.17)	*M* = 21.68 (2.55)	*M* = 21.98 (2.33)
**RHIQ (mean of items 1–3)**			
Synchronous & slow	*M* = 4.14 (1.70)	*M* = 4.03 (1.63)	*M *= 4.31 (1.57)
Asynchronous & slow	*M* = 2.70 (1.58)	*M* = 2.81 (1.46)	*M* = 2.91 (1.61)
Synchronous & fast	*M* = 4.50 (1.72)	*M* = 4.26 (1.56)	*M* = 4.42 (1.73)
Asynchronous & fast	*M* = 2.82 (1.45)	*M* = 3.05 (1.42)	*M* = 2.95 (1.51)
**Proprioceptive drift**			
Synchronous & slow	*M* = 1.22 (2.41)	*M* = 1.18 (3.04)	*M* = 0.90 (2.10)
Asynchronous & slow	*M* = 0.89 (2.36)	*M* = 0.80 (3.41)	*M* = 1.01 (1.87)
Synchronous & fast	*M* = 1.40 (2.48)	*M* = 0.74 (2.81)	*M* = 0.63 (2.94)
Asynchronous & fast	*M* = 0.66 (2.19)	*M* = 0.28 (2.35)	*M* = 0.01 (2.42)
**Comfort rating**			
Synchronous & slow	*Mdn *= 3.00	*Mdn* = 3.00	*Mdn* = 4.00
Asynchronous & slow	*Mdn* = 2.00	*Mdn* = 3.00	*Mdn* = 3.00
Synchronous & fast	*Mdn* = 2.00	*Mdn* = 3.00	*Mdn* = 3.00
Asynchronous & fast	*Mdn* = 2.00	*Mdn* = 2.00	*Mdn* = 3.00

### Sadness induction effects on proprioception

**Figure 2:**
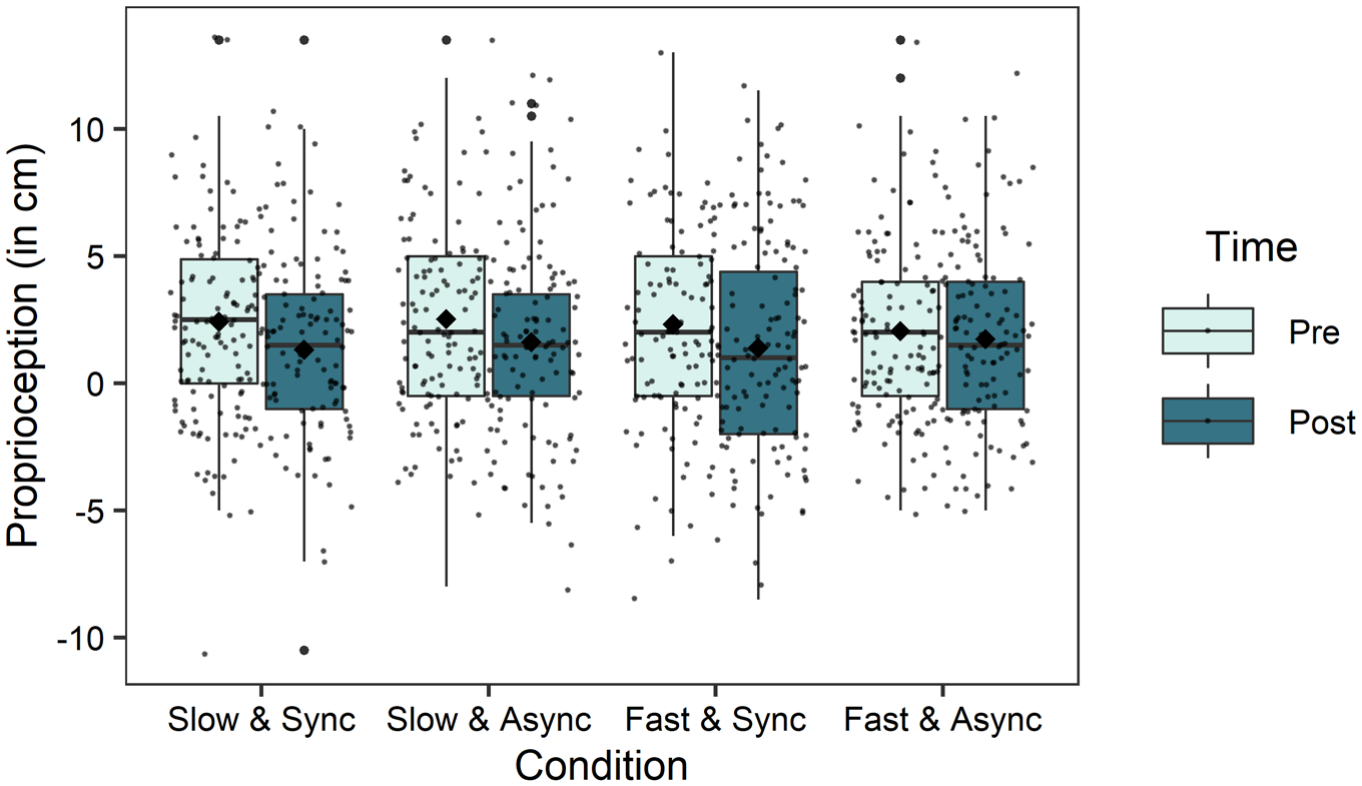
Visualization of the change in proprioception from pre to post stimulation in each condition, averaged across all groups. The rubber hand is positioned at −17.5 cm, accordingly, negative values indicate an estimated position between rubber hand and real hand. The means are indicated by the diamond shaped dots. The horizontal line represents the median. *Sync* Synchronous; *Async* Asynchronous.

Linear mixed-effects models with the predictors time, synchrony, speed, group and dissociative symptoms were calculated for the dependent variable proprioception. Non-significant random slopes and fixed effects were dropped, resulting in an intercept only model, which included only the main effect of time, showing a significant proprioceptive drift from pre- to post stroking (Fig. 2, Tables 3 and 4). No further main effects or interactions were significant, although descriptive data suggests greater drifts for slow and synchronous trials (Table 3). However, these differences did not significantly predict the outcome variable, possibly due to small effect sizes. 66.19% of the variance in proprioception was accounted for by the differences between the participants.

**Table 4:** Final linear mixed model for the dependent variable proprioception.

	Proprioception					
	Estimate	*SE*	*df*	*t*	*p*	*95% CI*
**Fixed effects**						
Intercept	2.33	0.29	137.02	7.97	** < .001**	1.78; 2.92
Time	− 0.81	0.14	854.00	− 5.82	** < .001**	− 1.07; − 0.55
**Random effects**						
σ^2^	4.73					
τ_00 Participants_	9.26					
ICC	0.66					
Marginal R^2^/conditional R^2^	0.01/0.67					

### Sadness induction effects on the rubber hand illusion questionnaire

**Figure 3:**
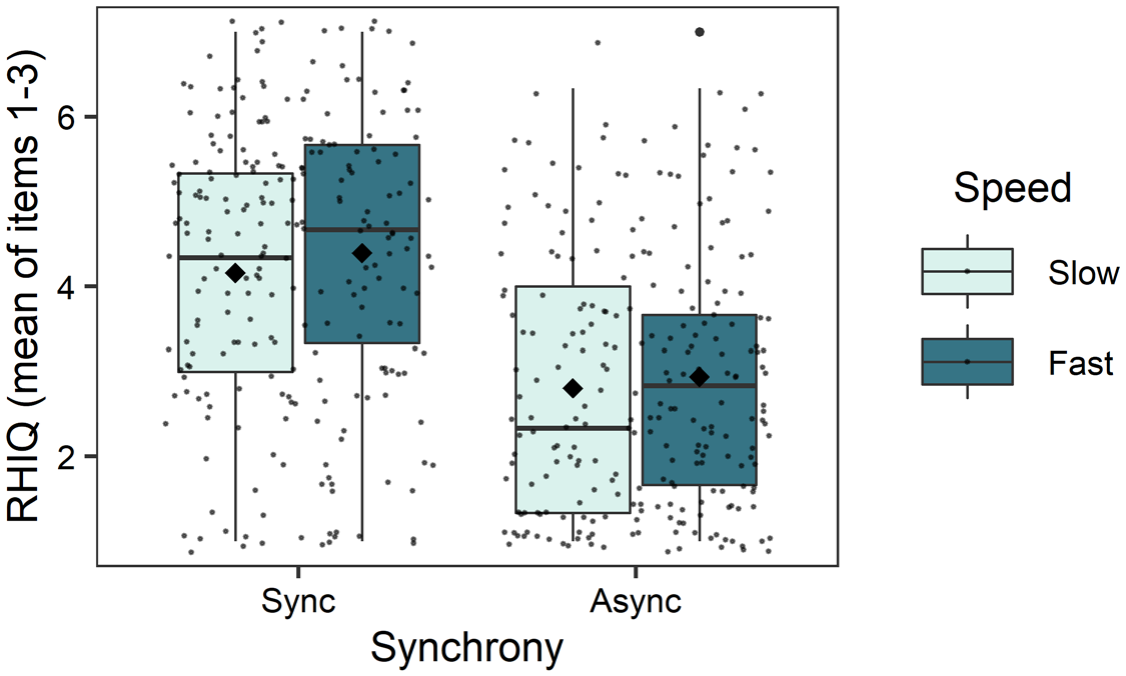
Differences between stroking conditions in the RHIQ, averaged across all groups. Higher values indicate a higher subjective illusion. The means are indicated by the diamond shaped dots. The horizontal line represents the median. *Sync* Synchronous; *Async* Asynchronous.

**Figure 4:**
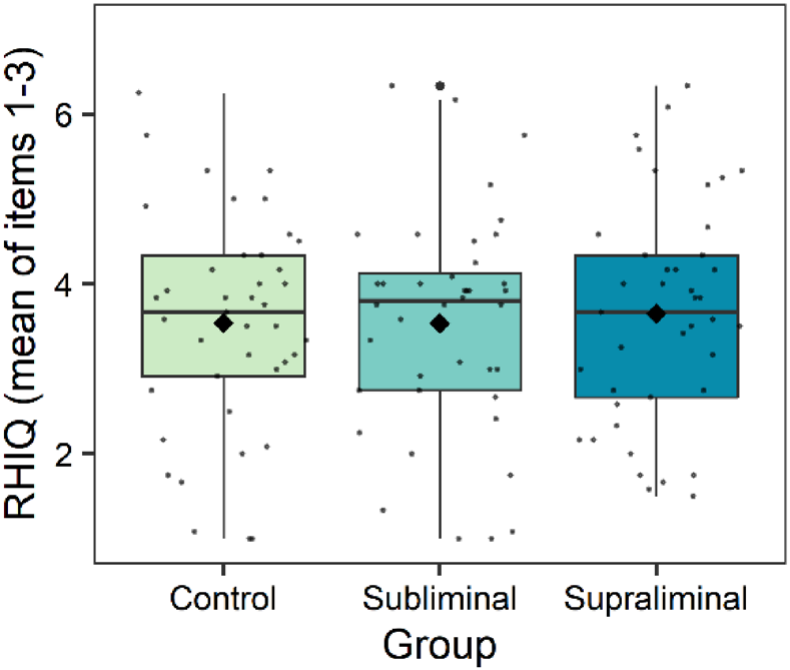
Group differences in the RHIQ, averaged across all four trials. The mean is indicated by the diamond shaped dots. The horizontal line represents the median.

**Figure 5:**
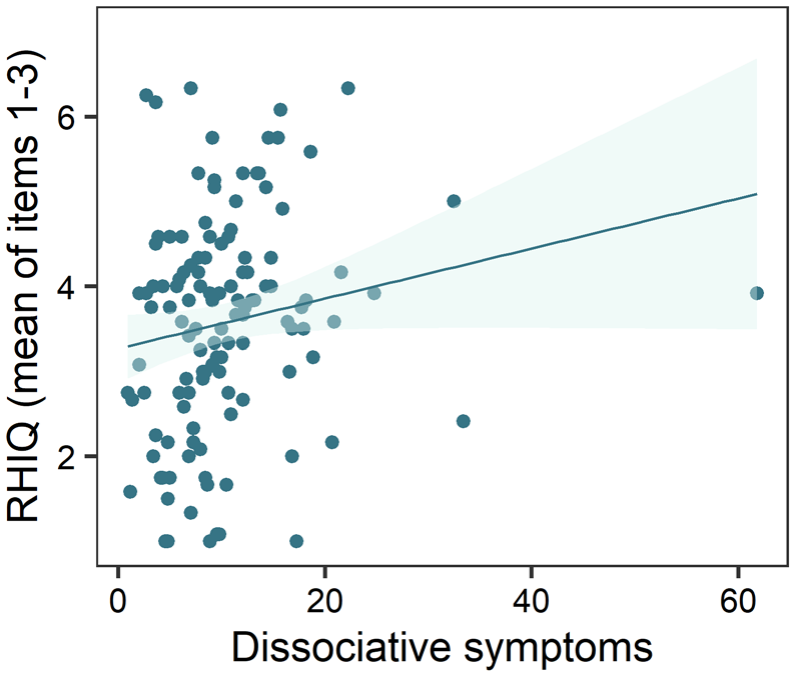
Regression graph for the main effect of dissociative symptoms on the mean RHIQ scores, averaged across all trials.

Linear mixed-effects models with the predictors synchrony, speed, group and dissociative symptoms were performed for the dependent variable RHI questionnaire (RHIQ). Non-significant random slopes and fixed effects were dropped, resulting in an intercept only model. The final model showed a significant difference in the RHIQ for the within subject factors speed and synchrony. Fast and synchronous stroking led to higher scores in the RHIQ compared with slow and asynchronous stroking (Tables 3 and 5, Fig. 3). There was no interaction between speed and synchrony, accordingly fast stroking seemed to elicit a stronger subjective illusion in both, synchronous and asynchronous trials (Fig. 3). A significant main effect of dissociative symptoms and a significant difference between groups was shown as well, but only between the subliminal (*M* = 3.54, *SD* = 1.30) and the supraliminal group (*M* = 3.65, *SD* = 1.32) (Tables 3, 5, Figs. 4 and 5).

**Table 5:** Final linear mixed model for the dependent variable RHI questionnaire.

	RHI questionnaire					
	Estimate	*SE*	*df*	*t*	*p*	*95% CI*
**Fixed effects**						
Intercept	3.43	0.23	182.15	14.60	** < .001**	2.98; 3.88
Speed	0.18	0.09	366.00	1.99	**.048**	0.002; 0.36
Synchrony	− 1.16	0.18	366.00	− 6.37	** < .001**	− 1.50; − 0.80
*Group*						
Con./Int.	− 0.11	0.17	169.30	− 0.64	.521	− 0.44; 0.23
Sbl./Spr.	− 0.87	0.27	169.30	− 3.24	**.001**	− 1.34; − 0.30
FDS	0.08	0.02	169.30	3.89	** < .001**	0.03; 0.11
*Synchrony × group*						
Synchrony × Con./Int.	0.02	0.13	366.00	0.17	.867	− 0.25; 0.28
Synchrony × Sbl./Spr.	0.31	0.21	366.00	1.44	.149	− 0.10; 0.72
Synchrony × FDS	− 0.02	0.02	366.00	− 1.61	.108	− 0.06; 0.007
*Group × FDS*						
Con./Int. × FDS	0.01	0.01	169.30	0.67	.506	− 0.02; 0.04
Sbl./Spr. × FDS	0.10	0.02	169.30	4.38	** < .001**	0.05; 0.14
*Synchrony × group × FDS*						
Synchrony × Con./Int. × FDS	0.004	0.01	366.00	0.43	.671	− 0.02; 0.03
Synchrony × Sbl./Spr. × FDS	− 0.04	0.02	366.00	− 2.33	**.021**	− 0.08; − 0.01
**Random effects**						
σ^2^	1.05					
τ_00 participants_	1.16					
ICC	0.52					
Marginal R^2^/conditional R^2^	0.25/0.64					

*Con.*  Control group, *Int.* Intervention, *Sbl*. Subliminal group, *Spr*. Supraliminal group.

**Figure 6:**
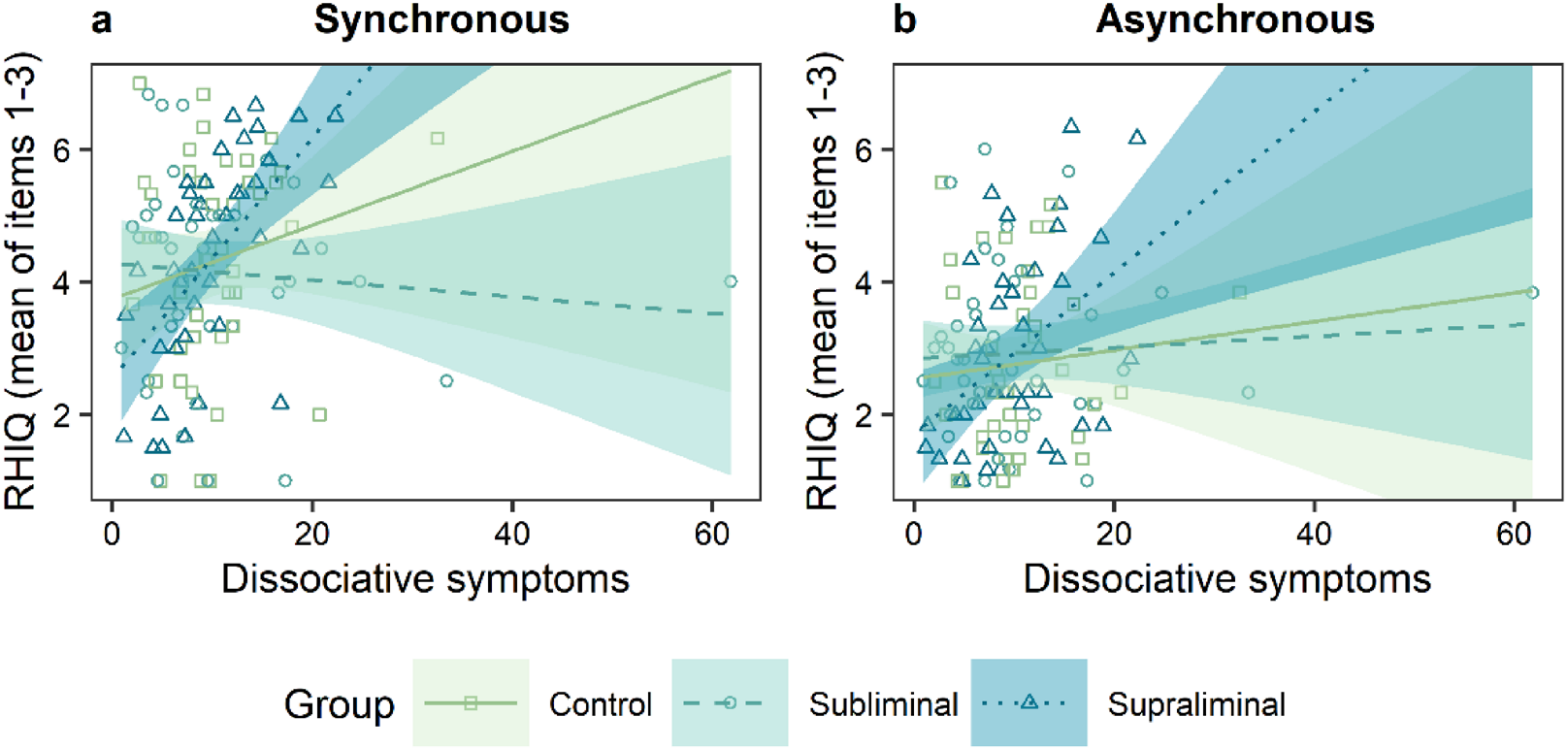
**(a)** Regression graph for the interaction effect of dissociative symptoms and group on the mean RHIQ scores, averaged across the synchronous trials. (**b)** Regression graph for the interaction effect of dissociative symptoms and group on the mean RHIQ scores, averaged across the asynchronous trials. Linear fitting was performed separately for each group.

Besides, the two-way interaction of subliminal vs supraliminal group with dissociative symptoms and the three-way interaction between subliminal vs. supraliminal group, synchrony and dissociative symptoms was significant (Table 5). Figure 6 demonstrates that compared to the subliminal group, people in the supraliminal group with a higher symptom burden experienced a stronger illusion than people with fewer symptoms. The comparison of Fig. 6a,b shows that the difference between the slopes of the supraliminal and subliminal group is steeper in synchronous trials. 52.46% of the variance in the RHIQ was accounted for by the differences between the participants.

### Control statements

In an ANOVA, no differences between the three experimental groups were found, *F*(2, 119) = 0.34, *p* = .714, *η*_*p*_^2^ = 0.006. Besides, a paired t-test was performed to check if there was a difference between control (mean of items 4–9) and illusion items (mean of items 1–3) in the RHIQ. Over all trials, the control items (*M* = 2.25, *SD* = 0.87) were found to be rated significantly lower than the illusion items (*M* = 3.57, *SD* = 1.29), *t*(121) = 12.57, *p* < .001, 95% CI [1.11; 1.53].

## Discussion

The present study aimed to investigate the effects of sadness on the RHI, while considering differences associated with emotion experience, such as dissociative symptoms and level of awareness of the emotion induction. In addition, stroking speed and synchrony were varied, as well. First, we found that the valence decreased more in the supraliminal group than in both other groups, while arousal was higher at the beginning and decreased over time. Our analysis also showed that regarding the subjective illusion, fast and synchronous stroking elicited higher illusion ratings than their counter-conditions. Moreover, the supraliminal sadness induction was associated with higher scores in the RHIQ compared to the subliminal condition. Contrary to our expectations, there was no difference between the subliminal emotion induction and the control condition. Additionally, a main effect of dissociation and an interaction between emotion induction style and dissociative symptoms was found, showing higher subjective illusion intensities in the supraliminal group compared to the subliminal group when participants had high scores on dissociative symptoms. A three-way interaction of dissociative symptoms, group (subliminal vs. supraliminal) and synchrony indicated that this effect was more pronounced in the synchronous conditions compared to the asynchronous conditions. Regarding proprioception, a significant drift from pre- to post illusion was detected. No group or stroking style differences and no effect of dissociative symptoms was found for the drift.

The finding that supraliminal sadness induction led to a stronger subjective illusion compared to the subliminal group, but no differences were found between both intervention groups and the control group, differs from the findings of previous studies investigating the relationship of emotions and the RHI^7,8^. The explanation given by Ehrsson et al.^7^ that the embodiment of external body parts under threat might be facilitated to enable more efficient body protection may not fully apply to other types of emotions that are less involved in body protection and are linked with lower levels of arousal^9,28^. Instead, Lambie and Marcel’s^12^ theory may provide a possible explanation why there were no significant differences between the control group and the intervention groups, but between subliminal and supraliminal emotion induction. According to Frijda, emotion experience can vary considerably, depending on the individual and the situation^11^. We therefore propose that the emotion induction itself is not essential, instead the mechanisms of focal attention involved in 2nd order emotional awareness, e.g. immersed vs. detached attentional mode and self- vs. world focus, may be relevant for the link between emotions and the sense of body ownership. For instance, supraliminal fear processing has been found to involve activity in the dorsal prefrontal and sensory brain areas, which play a role in attention direction and top-down processing^21^. Hence, attentional processes connected with 2nd order awareness could be crucial for the connection to body ownership. This may explain why no effects were found in the subliminal group, as this condition is considered to involve less 2nd order awareness^12^. As in the study by Winkielman et al.^20^ we did not observe an effect of subliminal induction on valence, supporting the lack of 2nd order awareness.

The importance of emotion experience becomes even more apparent when it comes to the interaction of group with dissociative symptoms. As mentioned earlier, emotion experience with self- or world focus and a detached attentional mode can lead to experiences of dissociation^11^. People with dissociative symptoms are known to respond to stressful or disturbing stimuli with depersonalization, derealization or flattening of the emotion experience^13,14^. They even experience lower physiological arousal, as measured by skin conductance and heart rate^29^. In our study, people with higher dissociative symptoms reported a higher subjective illusion. Our results are consistent with the study by Hirschmann and Lev Ari^16^, where patients suffering from dissociative PTSD experienced a stronger subjective RHI compared to healthy controls and patients with schizophrenia. The interaction between group, dissociative symptoms, and synchrony revealed that the connection between higher symptoms and a higher subjective feeling of embodiment was most prominent in the supraliminal group and in synchronous trials. Consequently, our results could be explained as follows: Presenting emotionally sad pictures in a supraliminal manner (involving 2nd order awareness) to subjects with higher dissociative symptoms could have led to a disembodiment reaction, resulting in a stronger illusion in the synchronous trials. This effect supports our hypothesis that the relationship of emotions and body ownership depends on features of emotion processing, like emotion experience^11,12^. This may also explain why some studies found large variances in the response to the illusion in samples of patients with dissociative symptoms^17^. Some patients might have experienced the illusion induction as aversive, while others did not. This could have influenced the extent to which patients experienced a detachment from the self. Further research is necessary to investigate this connection with regard to different emotions.

The effects of the supraliminal sadness induction were only found for the subjective feeling of embodiment, not for the proprioceptive drift. This could be due to different multisensory integration mechanisms: According to Rohde et al.^30^, the subjective illusion is based more on a visuo-tactile integration mechanism, whereas the proprioceptive drift results from a visuo-proprioceptive integration mechanism. Consequently, both measures of the RHI are not necessarily influenced equally by emotions. Sadness might have altered the processing of tactile signals, which is mainly relevant for the subjective feeling of ownership, but not so much for the proprioceptive drift. The sense of touch involves interoceptive processes which are strongly linked to emotions and also related to feelings of body ownership^31,32^. For instance, previous research has shown that the presentation of sad faces (low arousal), in a task that required subjects to count their own heartbeats, resulted in a stronger heartbeat evoked potential, which is a marker of interoceptive processes. In contrast, angry faces (high arousal) led to a reduction in heartbeat evoked potential and visual evoked potential, whereas positive faces did not elicit any effect^9,33^. Therefore, a connection between emotional arousal, interoception and the subjective ownership illusion is possible and should be further investigated in future studies.

Our expectations regarding the effects of the stroking conditions were partially confirmed by our data: Synchronous stimulation elicited a stronger subjective illusion compared to asynchronous stroking, but surprisingly, fast stroking led to a higher subjective illusion compared to slow stroking. Our results also demonstrated that slow and synchronous stroking had comforting effects that may have modified the effect of emotion induction. For instance, it has been shown that conflicting emotional information can lead to difficulties in emotional embodiment^34^. Besides, Riemer et al. argue that slow stroking in interaction with negative emotions might be perceived as less pleasant, which could also affect the illusion strength^26^. However, there was no interaction of group and stroking speed, speaking against this explanation. Another possibility would be that slow vs. fast stroking led to differences in arousal, which might explain the contradictory results. These considerations are quite speculative, therefore future studies should include a second SAM assessment after the RHI stimulation to confirm valence and arousal effects of different stroking styles. Besides, fast and synchronous stroking might be harder to discriminate and therefore more difficult to predict, which has previously been shown to lead to a stronger RHI^26^. In addition, it would be possible that subjects had difficulties discriminating strokes in fast, asynchronous trials, so that they may no longer have been perceived as asynchronous. These considerations may also partly explain the lack of differences in the proprioceptive drift between stroking conditions. Although descriptive data shows trends in the expected directions (greater drift for slow or synchronous conditions), no significant effect was found. This also supports the findings of Rhode et al., who found that the proprioceptive drift can also be present in asynchronous or vision only conditions and has different underlying multisensory integration mechanisms^30^. In addition, Crucianelli et al. also only found effects of slow vs. fast stroking on the subjective illusion, not the proprioceptive drift^5^.

Limitations of the study include the fact that our study sample was composed of healthy students, so the results on dissociative symptoms only refer to a subclinical population. Therefore, it would be interesting to investigate this connection in a patient sample. Besides, we did not include a questionnaire on state dissociation and therefore cannot evaluate whether the emotion induction resulted in self-reported dissociative symptoms in our experiment and whether these self-reports match the intensity of the RHI. However, it would be an interesting question for future research to investigate the effect of dissociative state tendencies on the magnitude of the illusion.

In addition, in previous research, a relationship between the subjective illusion and sensory suggestibility has been discovered^35^. It would be possible that subjects in the supraliminal sadness group are more prone to social desirability effects. For this purpose, the differences in control items between groups were analyzed, showing no differences. However, the existence of social desirability effects cannot be completely ruled out, as the use of control items as indicator of expectancy effects has recently been criticized^36^. To eliminate this possible explanation, the construction of more valid control items would be necessary.

Further limitations include the lack of control for emotional changes that might be induced by different stroking styles. Future research should measure the change in affect from pre- to post stroking to control for the impact of the comforting effect. Using physiological measures could also be helpful in this context. Besides, in the present study, the stroking was controlled only manually, which allows only limited control over the speed, the synchronicity and the reproducibility. Here, a technical device (such as a robot arm^30^), should be used in the future, as errors or irregularities in human movements are more likely to occur, especially in the asynchronous conditions.

It would also be interesting to directly compare emotions with high levels of arousal (like anger) to less arousing emotions (like sadness) to determine whether the differences between the present study and the study of Engelen et al.^8^ are due to different research designs or to the nature of the emotions.

Regarding practical implications, it could be very informative to examine the effects of body focused therapies (such as mindfulness-based stress reduction and progressive muscle relaxation) on the sense of body ownership^37,38^, as these approaches have experienced increasing popularity in recent years. Interventions such as mindfulness-based stress reduction aim to promote body awareness, resulting in decreased cognitive rumination about emotions, increased body related emotion experience and improved emotion regulation^39,40^. Thus, examining the link between emotions and the RHI in the context of a mindfulness intervention may provide a deeper understanding of the underlying mechanisms of mindfulness, paving the way for a promising therapy approach for disorders with disembodiment symptoms.

In summary, our results showed that compared to the subliminal group, supraliminal sadness induction led to a stronger feeling of body ownership. Surprisingly, we did not find the same results for the proprioceptive drift. This could be due to a distinction in the underlying multisensory integration mechanisms which might depend on the type of emotion induced. Besides, slow and fast stroking led to unexpected effects, namely, fast stroking led to a stronger subjective feeling of embodiment, possibly because of the comforting effect of slow stroking, differences in arousal or difficulties in discriminating between strokes. Furthermore, subjects with higher dissociative symptoms reported a stronger subjective illusion, especially after supraliminal sadness induction in the synchronous condition, indicating that disembodiment symptoms could arise in response to a sadness induction.

We conclude that various features of emotional processes are likely to play a role in feelings of body ownership, e.g. individual differences in emotion experience. The extent to which the subjective illusion and the proprioceptive estimation depend on the different forms of valence and arousal remains an open question. A more thorough investigation of this relationship may also provide insight into the underlying mechanisms of interventions based on mindfulness, which are strongly linked to body awareness and may be promising approaches to restore the balance between body and emotion in many disorders.

## Methods

### Participants

The required sample size was estimated using the program G*power^41^. In their second experiment, Engelen et al.^8^ found differences between an emotional sound, a no sound and a neutral sound condition with a medium effect size. Based on their results, we performed a power analysis for repeated measures analysis. Since a higher power was found for mixed models, the determined sample size should be sufficient^42^. Together with a power of 0.9 and an alpha value of 0.05, a sample size of 132 was estimated to detect the group effect. Following this estimation, 132 participants from the University of Regensburg (mainly students from applied movement science) were recruited. Ten participants had to be excluded because of outlier scores of more than 3 *SD* above/below the mean of the respective experimental group in the RHIQ or in the proprioceptive drift^35^ or because they wore non-removable bracelets or had injuries on the relevant left arm. Of ten excluded participants, four had +/− 3SD outliers, five subjects wore non-removable jewelry and two had injuries (for one person two criteria applied). Consequently, the remaining sample size was *N* = 122. Overall, the sample population consisted of 86 females (age: *M* = 21.24, *SD* = 1.87) and 36 males (age: *M* = 22.83, *SD* = 2.94), ranging in age from 18 to 32 years.

Written informed consent was obtained from all participants in advance. Following study completion, participants were briefed on the study background. The present study was performed in accordance with the Declaration of Helsinki and was approved by the ethics committee of the University of Regensburg (protocol number: 18-1204-101).

## Materials

### Sadness induction

At the beginning of each trial, participants watched a series of pictures on a computer screen. The stimuli sets consisted of either 24 sad or 24 neutral pictures, retrieved from the International Affective Picture System (IAPS)^43^. Both sets were composed of pictures from multiple categories, e.g. people of all ages and ethnicities, nature or indoor settings, buildings and landscapes or social contexts^44^ and were selected based on their ratings on a 9-point SAM scale (sad: mean valence = 2.74, mean arousal = 4.74; neutral: mean valence: 5.06, mean arousal = 3.29^43^). The subjects were randomly assigned to three conditions: One group saw pictures from the emotionally sad picture set for a duration of 2000 ms per stimulus, so a supraliminal perception of the picture was possible. The second group saw the same sad stimuli, presented subliminally for 30 ms each, masked by a 2000 ms presented neutral picture. Finally, the third group only watched neutral pictures, each shown for 2000 ms^44^. During each trial, the pictures were presented twice in a random order, so a total of 48 stimuli were shown before each RHI stimulation condition. Between the stimuli, a 500 ms fixation point was shown. Every eight stimuli there was a short 6000 ms break. Beforehand, participants were asked to sit in front of the computer and fixate their eyes on the screen. They were instructed to look at the pictures for as long as they were presented and to return their gaze to the fixation point whenever it was shown.

### Self-assessment manikin (SAM)

To measure the effect of the sadness induction, we recorded the mood of the participants in the beginning and after all four picture trials. For this purpose, we used a paper-pencil version of the SAM scale, which assesses valence, arousal and dominance in a non-verbal way. For each dimension, participants were asked to rate their emotional state on a 9-point Likert scale (1 indicates being unhappy/unaroused/controlled; 9 indicates being happy/aroused/controlling), which corresponded to five figures and the spaces between the figures^27^. Participants were instructed to choose which one of the figures most closely described their emotional state. If they could not decide between two figures, they could also tick the number in the between them. In the past, the SAM scale has frequently been applied in connection with the IAPS. In this combination, it was highly correlated with other tests and physiological measures, like skin conductance response, cardiac acceleration, viewing time, interest ratings and muscle activity^27,45^.

### Rubber hand illusion stimulation

**Figure 7:**
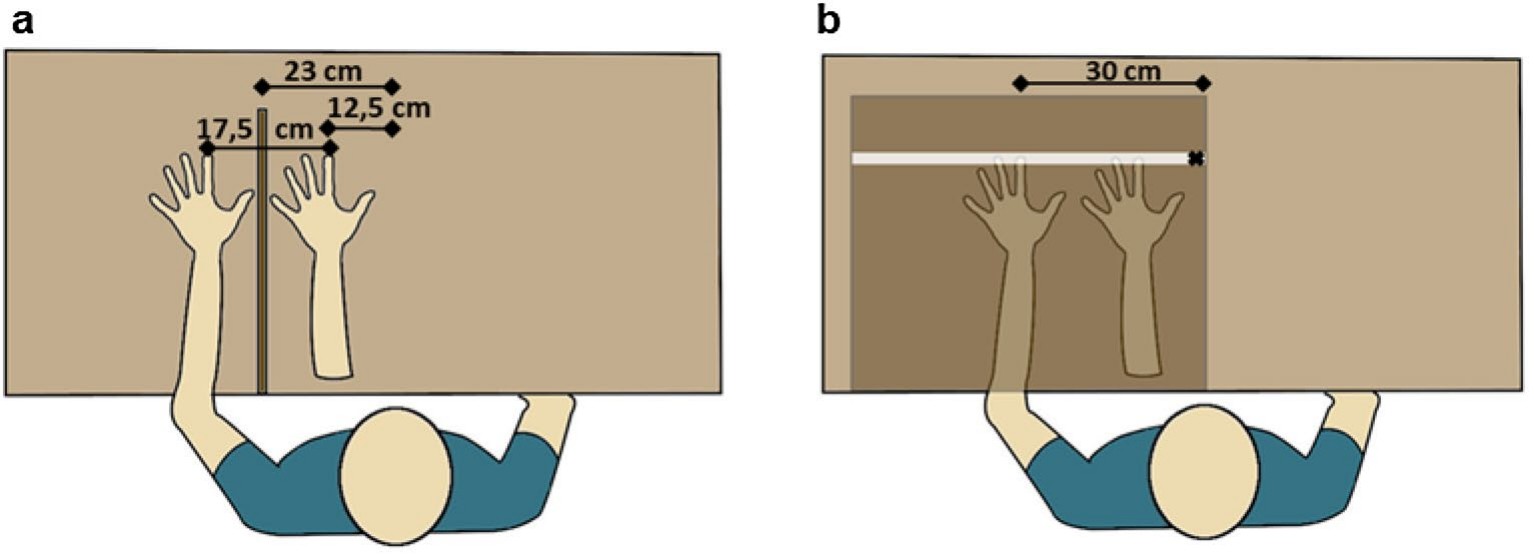
Illustration of the experimental setup. (**a)** RHI stimulation setup. (**b)** Proprioception measure, applied using a wooden box with a tape measure.

Throughout the stimulation, participants placed their real left arm on a table behind a wooden screen (40 cm × 1.50 cm × 55 cm), so it was hidden from view. The screen was installed 23 cm to the left of the body centre of the participants, which corresponds to the average shoulder length of males and females in Germany (males: 48 cm, females: 43.50 cm, both: 45.75 cm—shoulder distance from body centre: 22.87 cm)^46^. On the right side of the screen, a rubber arm (Killerink, Liverpool, UK) was positioned visibly, with a distance of 17.50 cm between both index fingers (Fig. 7a)^3,47,48^. During the entire experiment, participants wore a customary black hairdresser’s gown to cover up the visible parts of the subject’s own left arm and the stump of the rubber arm. At the beginning of each trial, participants were instructed to simply look at the rubber arm for 15 s^5^. Subsequently, the stimulation was performed with two identical, synthetic, 30 mm rouge brushes. The experimenter stood at the opposite side of the table and stroked the real hand and the rubber hand either in synchrony or asynchrony, combined with slow stroking (approx. 3 cm/s) in one half of the trials and fast stroking (approx. 30 cm/s) in the other half of the trials^4^ (Supplementary Video S1). The stimulation lasted 60 s and was applied to the forearm, as it has been done in other studies comparing pleasant and unpleasant touch, e.g. Crucianelli et al.^5^. Speed was controlled visually using a timer and marked sections on the table, which allowed for slight variations in stroking speed. Participants were instructed to look at the rubber arm for the entire duration of the stimulation.

### Proprioceptive drift

To measure the change in the proprioceptive estimation from pre to post-stimulation, a wooden box (49.50 cm × 74.50 cm × 10.50 cm) was placed over the real- and the rubber arms with the lower right corner of the box positioned at the body center (Fig. 7b). A white tape on the top edge of the box indicated the distance from the body to the tip of the index finger. Participants were asked to place their right index finger above the marked X on the tape and to slide their finger along it with closed eyes until they reached the position below which they believed their left index finger to be^8^. Subsequently, the distance between the actual real finger position and the estimated position was calculated.

### Rubber hand illusion questionnaire (RHIQ)

Following the post-stimulation proprioception measure, a questionnaire was given to the participants. It included a German translation of the RHIQ^2^ (Supplementary Table S1). The RHIQ consists of 9 items and is measured on a 7-point Likert scale, ranging from “strongly disagree” (1: “− − −”) to “strongly agree” (7: “+ + +”). In the past, the first three items of the RHIQ were found to be particularly suitable in measuring the strength of the illusion, while items four to nine were used as control items^2,4,30^. Internal consistencies for the illusion (α = 0.80–0.88) and the control items (α = 0.70–0.75) were reasonable. Therefore, a composite score of the illusion items (1–3) was used for the analysis and referred to as “rubber hand illusion questionnaire” or RHIQ.

### Comfort rating

In order to assess whether the stroking was perceived as comforting, we created an item (“I felt comforted by the touch”) with a 6-point Likert scale ranging from 1 = “totally disagree” to 6 = “totally agree”.

### FDS questionnaire

The FDS (Fragebogen zu dissoziativen Symptomen) is a German adaption of the American Dissociative Experience Scale (DES)^49,50^. It was conceptualized as a screening instrument with 44 items and an 11-point Likert scale, ranging from 0% (never) to 100% (always) in steps of 10%. It indicates the severity of four different dissociative symptoms (amnesia, absorption, derealisation and conversion). The internal reliability of the FDS was fairly high in the literature (α = 0.93), as well as the retest-reliability (14 days: .82)^50^. In the present study, we also found an internal consistency of α = 0.93.

### Procedure

**Figure 8:**
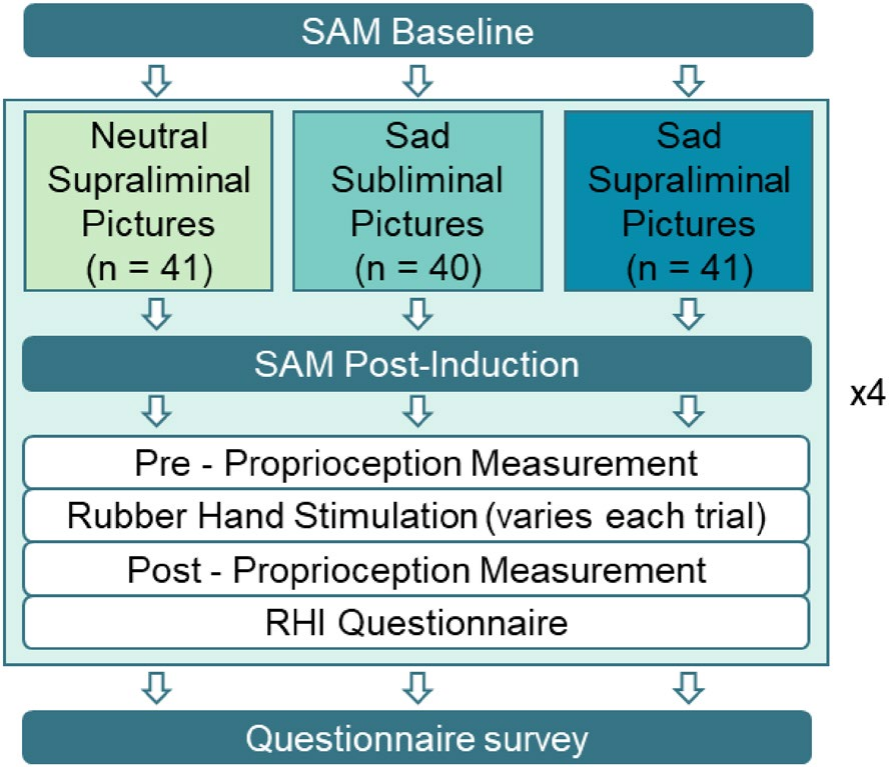
Experimental procedure. The tasks displayed in the light blue box were repeated four times, each time with a different stroking condition (e.g. asynchronous × slow touch).

Before the start of the experiment, we conducted a baseline assessment of the SAM scale to determine the participants’ initial level of valence, arousal and dominance. We then conducted four experimental trials. Every trial began with a picture series: Participants either viewed neutral pictures (control group), supraliminally presented sad pictures (supraliminal group) or subliminally presented sad pictures (subliminal group). Afterwards, the SAM scale was completed again to assess the affective reactions to the pictures (Fig. 8).

The second part of each trial consisted of the RHI stimulation. We conducted four trials to measure the reactions to different stimulation conditions (Fig. 8). For this purpose, we combined either synchronous or asynchronous with slow or fast stroking, resulting in four conditions: Synchronous × slow stroking, asynchronous × slow stroking, synchronous × fast stroking and asynchronous × fast stroking. The order of the conditions was counterbalanced among groups, so that 25% of each experimental group received the same order of trials. Before and after the stimulation, the proprioceptive estimation was recorded. In addition, the subjective illusion questionnaire and the comfort rating were completed following the post-proprioception measurement. After the experimental part of the study, participants completed computerized questionnaires on dissociative symptoms (FDS) and their demographical background.

### Statistical analysis

Statistical analyses were conducted using R (version 4.0.3)^51^. First, it was checked whether our three groups led to the presumed mood effects. Consequently, cumulative link mixed models from the package “ordinal”^52^ with equidistant thresholds were calculated for the SAM dimensions valence, arousal and dominance with trial (simple contrast coded with reference to the baseline) as within subject factor and the experimental group (Helmert contrast coded: control vs. intervention (subliminal & supraliminal) and subliminal vs. supraliminal) as between subject factor.

Subsequently, another cumulative link mixed model with equidistant thresholds and the predictors synchrony (synchronous vs. asynchronous), speed (slow vs. fast) and group (Helmert contrast coded as described above) was calculated to analyze whether participants judged the comforting effect of the stimulation differently depending on stroking style and group affiliation.

The next analysis was performed to check for differences in the illusion between the subliminal, the supraliminal and the neutral group and to examine if the different stroking styles had the presumed effects. For the two measures of the illusion, linear mixed models (package “lme4”^53^) with maximum likelihood estimation and the wrapper optimix were calculated^54,55^. Normality, linearity and homoscedasticity were checked visually and revealed no deviations. As predictors synchrony (synchronous vs. asynchronous), speed (slow vs. fast), group (Helmert contrast coded as described above) and dissociative symptoms were entered into the model for the RHI questionnaire. For the proprioceptive drift, the predictor time (pre vs. post stimulation) was also added.

Model selection for both, the cumulative link mixed models and the linear mixed models was performed according to Barr et al.^56^ and Matuscheck et al.^57^: Initially, the maximal model was formed for each dependent variable, which included a random intercept and random slopes for all fixed effects^56^. Then, non-significant variance components were removed stepwise and the fit of the new model was compared to the previous model using likelihood ratio test. In case of a loss in goodness of fit with p < .200^57^, model reduction was stopped, unless the model continued to show convergence issues. Subsequently, non-significant fixed effects were excluded from the model based on the likelihood ratio test. In case of a significant deterioration in model fit (p < .05), the model complexity reduction was stopped and the resulting final model was reported. For the ordinal dependent variables, Wald confidence intervals were calculated^52^, whereas bootstrapping with 1000 simulations was performed for numerical dependent variables^55,58^.

Finally, to examine if the control items (items 4 to 9) of the RHIQ differed significantly between groups, an ANOVA was performed. In addition, to check whether the control items differed significantly from the illusion items (items 1 to 3) of the RHIQ, a dependent t-test was calculated between the mean of the control items and the mean of the illusion items.

## Supplementary Information

Supplementary Information.

## Data Availability

The datasets generated during and analyzed during the current study are available in the [Open Science Framework] repository [https://doi.org/10.17605/OSF.IO/SMRB4].
