# Supplementary material for: The effects of subliminal or supraliminal sadness induction on the sense of body ownership and the role of dissociative symptoms
Source: Sci Rep. 2021 Nov 15;11:22274. doi: 10.1038/s41598-021-01039-2 (PMC8592987; doi:10.1038/s41598-021-01039-2)
Supplement: Supplementary file 1 — Supplementary Information. [file 41598_2021_1039_MOESM1_ESM.zip › Supplementary Information.docx]

**Supplementary Information: Legend**

Table S1. RHI questionnaire translation.

Video S1. Demonstration of the different stroking style procedures (synchronous & slow, asynchronous & slow, synchronous & fast, asynchronous & fast).
