# Supplementary material for: The effects of subliminal or supraliminal sadness induction on the sense of body ownership and the role of dissociative symptoms
Source: Sci Rep. 2021 Nov 15;11:22274. doi: 10.1038/s41598-021-01039-2 (PMC8592987; doi:10.1038/s41598-021-01039-2)
Supplement: Supplementary file 1 — Supplementary Information. [file 41598_2021_1039_MOESM1_ESM.zip › SupplementaryTable_S1.docx]

| German translation | Original items of Botvinick and Cohen (1998) |
| --- | --- |
| 1. Es schien so, als würde ich die Berührung des Pinsels dort fühlen wo sich die Gummihand befand. | 1. it seemed as if I were feeling the touch of the paintbrush in the location where I saw the rubber hand touched. |
| 1. Es schien so, als ob die Berührung, die ich empfand, durch das Streicheln der Gummihand entstehen würde. | 1. It seemed as though the touch I felt was caused by the paintbrush touching the rubber hand. |
| 1. Es fühlte sich so an, als ob die Gummihand meine eigene Hand wäre. | 1. I felt as if the rubber hand were my hand. |
| 1. Es fühlte sich so an, als ob sich meine eigene Hand nach rechts (in Richtung der Gummihand) bewegen würde. | 1. I felt as if my (real) hand were drifting towards the right (towards the rubber hand). |
| 1. Es fühlte sich so an, als ob ich mehr als eine linke Hand/Arm hätte. | 1. It seemed as if I might have more than one left hand or arm. |
| 1. Es fühlte sich so an, als ob die Berührung, die ich empfand von einer Stelle zwischen meiner realen Hand und der Gummihand herkam. | 1. It seemed as if the touch I was feeling came from somewhere between my own hand and the rubbser hand. |
| 1. Es fühlte sich so an, als ob meine eigene Hand zu Gummi werden würde. | 1. It felt as if my (real) hand were turning “rubbery”. |
| 1. Es sah so aus (visuell), als ob sich die Gummihand nach links (in Richtung meiner eigenen Hand) bewegen würde. | 1. It appeared (visually) as if the rubber hand were drifting towards the left (towards my hand). |
| 1. Die Gummihand begann meiner eigenen Hand ähnlich zu sehen, hinsichtlich ihrer Form, Hautfarbe, Sommersprossen oder anderer Merkmale | 1. The rubber hand began to resemble my own (real) hand, in terms of shape, skin tone, freckles or some other visual features. |

Table S1. RHI questionnaire translation
